# The Differential Effects of Physical Activity Calorie Equivalent Labeling on Consumer Preferences for Healthy and Unhealthy Food Products: Evidence from a Choice Experiment

**DOI:** 10.3390/ijerph18041860

**Published:** 2021-02-14

**Authors:** Xiaoke Yang, Yuanhao Huang, Mengzhu Han, Xiaoting Wen, Qiuqin Zheng, Qian Chen, Qiuhua Chen

**Affiliations:** 1 College of Management, Fujian Agriculture and Forestry University, Fuzhou 350002, China; 2171573003@fafu.edu.cn (X.Y.); 1181542003@fafu.edu.cn (M.H.); 1191565007@fafu.edu.cn (X.W.); 2191573001@fafu.edu.cn (Q.Z.); 000q091001@fafu.edu.cn (Q.C.); 2 School of Business, Renmin University of China, Beijing 100089, China; 2019000720@ruc.edu.cn

**Keywords:** PACE labeling, food choice, FTP, unhealthy foods, healthy foods

## Abstract

*Background*: Since numerical calorie labels have limited effects on less-calorie food ordering, an alternative called physical activity calorie equivalent (PACE) labels, which exhibit calories using visible symbols and the minutes of exercise to burn off the calories, may be more effective in reducing calories ordered. *Methods*: By using a choice experiment (CE) approach, the aims of this study were to estimate the effects of PACE labels on consumer preferences for healthy and unhealth food. Red date walnuts and potato chips were used as the representatives of healthy and unhealthy foods respectively in this study. Moreover, future time perspective (FTP) is an individual trait variable of consumers, which has been recognized as a significant driver of healthy behaviors. We also included FTP into the interaction with PACE labels. *Results*: Firstly, the results were opposite between the healthy and unhealthy food groups. Respondents showed significantly more positive attitudes toward red date walnuts (i.e., healthy food) with PACE labels, while they showed significantly more negative preferences for chips (i.e., unhealthy food) with PACE labels. Secondly, people with higher FTP are preferred red date walnuts with PACE labels, while PACE labels on chips could undermine the preferences of respondents with higher FTP. Thirdly, we found that women (vs. men) were less inclined to choose healthy food with standard calorie labels and labels showing the minutes of running to burn off the calories, as well as that the elderly (vs. younger) people in the healthy food group preferred the labels showing the minutes of running to burn off the calories. People with a higher body mass index (BMI) were reluctant to purchase walnuts with the information about the minutes of walking. *Conclusions*: Results from this study showed that PACE labels have significant effects on consumers’ preferences for food products.

## 1. Introduction

The global obesity epidemic has gained a lot of attention in recent decades due to its links with some chronic diseases, such as type 2 diabetes, dyslipidemia, cardiovascular disease, and periodontal disease [1,2,3]. The prevalence of overweight in China has increased steadily between 2002 and 2020 (from 14.7% to 34.4% for adolescents), the average weight of men and women were 69.6 and 59.0 kg respectively [4]. The consequences of obesity are not only chronic diseases, but also a greater burden on governments in terms of expenditures on the chronic diseases caused by the obesity [5].

Two major causes of obesity across all populations are unhealthy eating and inadequate exercise [6]. Governments are exploring numerous strategies to prevent the obesity epidemic, with the dual aims of preventing excess energy intake and promoting healthier food choices [7,8]. Nudge interventions are adopted as a spur to healthier eating that has arouse some interest from policy-makers and researchers [9]. Food labeling is a nudging tool to alter people’s behavior through providing information about foods [9,10], which is generally adopted by many countries [11]. For example, policymakers have required food manufacturers to provide nutritional information (including calorie information) on packaged foods [12]. In the United States, there is legislation on calorie labeling on all menu forms in chain restaurants that are used to encourage lower-calorie choices [13]. However, studies on the effects of calorie labels do not reach a consensus [14,15,16,17,18]. According to Viera et al. [19], only kcal information on the calorie labeling would fail to promote the purchase of lower-calorie foods. Standard calorie labeling is too detailed to understand [20]. Consumers spend an average of six seconds when scanning food products before making purchase decisions [21]; simple labeling is a more readily understandable manner that might be more effective in making changes toward healthy diets [19,22]. Compared to numerical calorie labels, labeling with symbols has a much greater influence on healthier food purchases because it is understood by consumers [23].

Physical activity calorie equivalent (PACE) labels, which were proposed by the Royal Society for Public Health [24], which exhibits the miles or minutes of different sports needed to burn off the calories based on the consumption of a certain food item [25]. A PACE label exhibits the calories in visible forms [26]. Researchers want to estimate whether PACE labels influence low-calorie purchasing behaviors. Results appear contradictory for the effects of PACE labels [16]. The results of Viera and Antonelli [27] showed that parents who were shown the calories as well as the minutes and miles ordered fewer calories for their children, and were also more inclined to get their children to exercise. Dowra et al. [28] also showed that people ordered fewer calories from menus with exercise information, and 82% of participants preferred menus with physical activity labels over those with calorie information only or no information. Similar conclusions have been drawn by several works in the literature [26,28]. However, the opposite results were shown in a study by Shah et al. [29], who found that there were no differences in between PACE-labeled and calorie-labeled food choices among Hispanic consumers. Similarly, the calories ordered when using PACE labels and calorie labels did not show a statistically significant difference in a meta-analysis [16]. A non-hypothetical study conducted in three cafeterias demonstrated that PACE and calorie labels were able to promote consumers to purchase fewer calories, but the difference between PACE and calorie labels was not significant [30]. Taken together, the effects of PACE labels are still open to debate.

Seyedhamzeh et al. [16] mentioned that differences in types of foods and forms of PACE labeling may have caused the inconsistent results across these studies. For example, PACE labels could reduce prospective food consumption of familiar snack foods, while this effect was not presented in the unfamiliar snack groups [31]. Talati et al. [32] has estimated three front-of-pack (FoP) labels among four food products of varying healthiness, results showed that the Daily Intake Guide and Multiple Traffic Light labels could enhance the favorable evaluations of unhealthier product (cookies), with little impact on healthy product (yoghurt). Similarly, a study by Lee et al. showed that organic labeling has opposite effects on food consumption between vice (unhealthy)and virtue (healthy) food [33]. In conclusion, effects of different FoP labels should be addressed in specific food products. As for PACE labels, researchers usually focused on energy-dense unhealthy foods in the aforementioned literature. However, some foods with high energy contents have been ignored when determining the effects of PACE labels. For example, nuts are recognized as a healthy food choice, as they are good sources of many nutrients, but they are also high in energy and fat [34]. Thus, the question remains whether PACE labels influence consumer preferences for all energy-dense foods, including unhealthy and healthy alternatives, and whether there is any difference between the PACE labeling on preferences for those two kinds of food? Moreover, prior studies have also demonstrated a discrepancy between different contents of physical activities (comparisons between different sports, or miles vs. minutes) [16,28]. Therefore, it is important to take forms and different kinds of food into consideration to determine the effects of PACE labeling. Moreover, socio-demographics of consumers need be taken into consideration when choosing products [35]. In a systematic review on PACE labels, Seyedhamzeh et al. [16] found that the participant characteristics (age, gender, BMI) were contained in relative literature about PACE labels. Thus, age, gender and BMI were selected as the representative socio-demographic variables in this study, these variables also were examined in a previous study [36]. Compared with other approaches, choice experiment (CE) can measure decisive attributes of consumers’ preferences for the product accurately and provide much more information regarding when detecting consumers’ preferences [37], which is suitable for this study to detect consumers’ preferences for healthy and unhealthy foods with PACE labels, and taking different physical activities and socio-demographics into consideration. To the best of our knowledge, this is the first study that research on the preferences for PACE labeling for the first time. Taken together, this study values consumers’ preferences PACE labels (based on walking and running) of healthy and unhealthy foods using the CE approach.

When it comes to research on healthier food purchasing, the future time perspective (FTP) is an individual trait variable of consumers that might play a significant role in healthy behaviors [38]. According to Hall et al. [38], people with a future-oriented time perspective are more inclined to consider long-term benefits than who are present-oriented; in other words, people who put more emphasis on profound benefits rather than subtle short-term costs are more engaged in health-protective behaviors (e.g., exercise and healthy diets). Meanwhile, people with higher future time perspectives tend to delay gratification [39] and have a higher subject awareness of health [40]. Previous related research focused on the time perspective and showed that consideration of future consequences was a significant psychological determinant of consuming organic foods [41,42]. Onwezen et al. [43] mentioned that time perspective can stimulate healthy consumption of food. However, to the best of our knowledge, no study to date has analyzed the specific relation between FTP and food choices in the context of PACE labels.

The aims of this study were triple: (a) to estimate the effects of different forms of labeling (none, standard calorie label, PACE label with minutes of walking, and PACE label with minutes of running) of an unhealthy product and a healthy alternative (potato chips and red date walnuts); (b) to determine the FTP of consumers and the interaction effects between FTP and PACE labels; (c) to measure the interaction effects of socio-demographic indicators (age, gender and BMI) and PACE labels on consumers’ preferences for foods.

## 2. Materials and Methods

In previous studies on consumer preferences for PACE labels, participants were assigned to different label information conditions and were asked to select the provided foods, furthermore, researchers estimated the differences in evaluations of provided foods or calorie ordering between groups under different label conditions [26,44,45]. In the current study, we designed different label scenarios in healthy and unhealthy foods groups through a CE approach to estimate consumer preferences for PACE labels. CE has been adopted by many recent studies to determine consumer preferences and willingness to pay (WTP) for products [46,47,48]. CE can narrow the bias by grouping attributes into different components [49]. Beyond that, CE is more accurate in estimating conjoint effects, as the approach is less influenced by social desirability bias [50]. This study estimated the interaction effects between FTP and different labels by using a CE.

### 2.1. Attribute Selection

**Figure 1:**
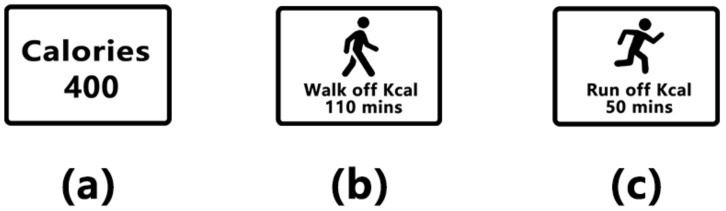
(**a**) The kcal label; (**b**) label showing the minutes of walking needed to burn off the calories; (**c**) label showing the minutes of running needed to burn off the calories.

The efficiency and accuracy of the CE approach were determined according to the chosen attributes [49], which means that attributes were selected based on the policy implications and if they had a significant influence on consumer preferences [51]. In this study, we chose three labels (standard kcal label, a label showing the minutes of walking needed to burn off the calories, and a label showing the minutes of running needed to burn off the calories) and a condition without a label (see Figure 1). The forms of PACE labels were referred to prior literature [52,53]. The total calories of the two products were calculated based on the original calorie information printed on the backs of their respective packages, and the calories of walking and running burn off per hour were refer to Keep App, which is a professional health app that contains data collected about calories of different sports burn off per hour. In our experiment, red date walnuts and potato chips were used as representatives of healthy and unhealthy foods respectively. A 127 min walk and a 58 min run were required to burn off the 465 kcal contained in the red date walnut product, and a 110 min walk and a 50 min run were required to burn off the 400 kcal contained in the potato chip product.

According to the review by Thow et al. [54], the effective rates of taxes or subsidies on food range from 10% to 20%. Considering the two kinds of food that we chose are cheap in real markets, a 30% tax rate was used to avoid unobvious price changes. Finally, the price attribute was set at four levels: the market price and taxes of 10%, 20%, and 30%. The details can be seen in Table 1.

### 2.2. Experimental Design

The accuracy of a CE depends on the structure of choice sets, which means that the designed choice sets should explain the maximum variance of the attributes while minimizing the random errors [55]. Many non-hypothetical studies have employed an opt-out to imitate the realistic market conditions [56]. However, several studies have mentioned that an opt-out option is not appropriate for all scenarios [57,58]. An opt-out option has no influence on marginal WTP and causes greater unobserved heterogeneity [59]. Thus, the opt-out option was not adopted in this study.

**Figure 2:**
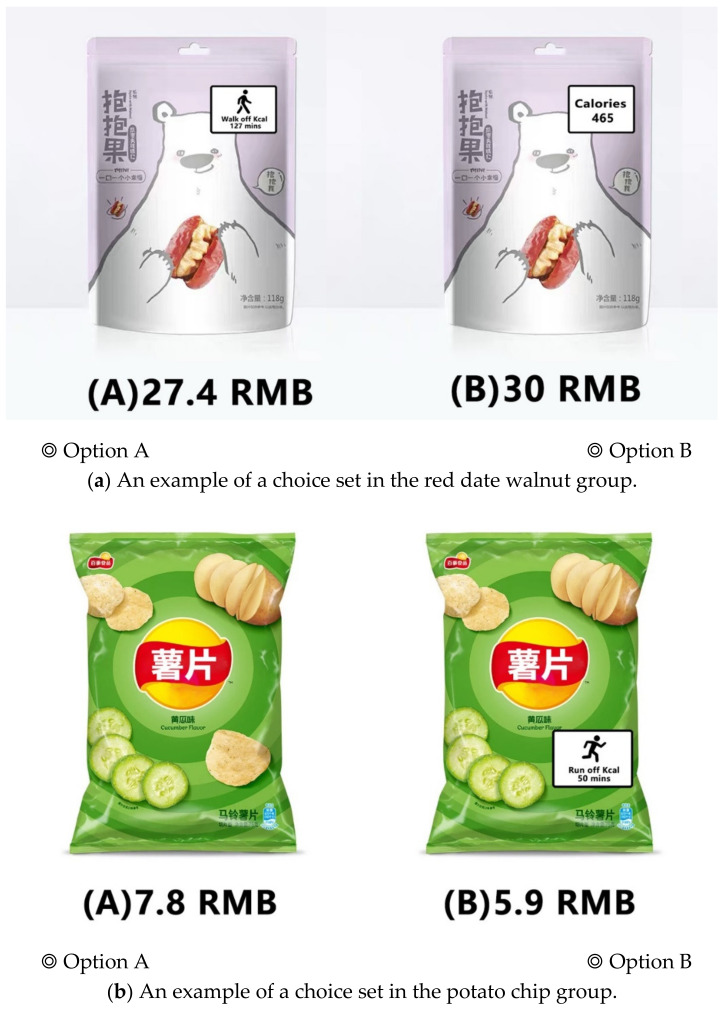
Examples of the choice sets in the choice experiment (CE).

A full factorial design in the Negene 1.1 software (ChoiceMetrics, New South Wales, Australia) was based on the two selected attributes and four levels of each attribute, and as for the two options we designed, which generated (4 × 4)^2^ = 256 choice sets. It was impossible for the respondents to evaluate all of the choice sets. A fractional design was determined to be appropriate for this study, as it can maintain the efficiency of profiles while minimizing the number of tested choice sets [60]. Finally, a D-optimal design was used to generate 20 choice sets with a D-efficiency of 84.52%, D-error of 0.07, and A-error of 0.07. The 20 choice sets were randomly divided into three blocks, which respectively contained 7, 7, and 6 choice sets. Each choice set contained two alternatives (see Figure 2).

The FTP of consumers was measured with an improved Zimbardo Time Perspective Inventory derived from a study by He et al. [61]. In particular, this improved version is more suitable for Chinese consumers [61], and includes five items: “I usually complete my plan step by step and on time”; “Before I play tonight, I will finish tomorrow’s tasks”; “When I want to accomplish something, I set a goal and take measures to reach it”; “As long as they help me, I will persist in completing these difficult and boring tasks”; “I always fulfill my promises to friends and superiors on time.” All of the items were evaluated on a five-point Likert scale, from 1 for “strongly disagree” to 5 for “strongly agree”.

In order to confirm whether consumers could distinguish between the health levels of red date walnuts and potato chips. We tested this question with two items, which referred to the study by Naylor et al. [62]: “The food in the questionnaire is healthy,” and “The food in the questionnaire contains numerous nutrients.” These two items were scored based on a five-point Likert scale, from 1 for “strongly disagree” to 5 for “strongly agree”.

### 2.3. Data Collection

Before we conducted the experiment, a pre-test (N = 56) was conducted in the School of Business in Renmin University of China. We sent designed stimuli (red date walnuts and potato chips with different PACE labels) to 56 undergraduates through E-mail, and asked them to reply whether they could clearly distinguish the differences of PACE labels with running or walking. Finally, we got 53 answers that expressed they can understand the meaning of PACE labels, and other 3 students didn’t reply to us. Results indicated that respondents can correct understanding of the stimuli in this study.

We conducted the experiment through a professional online survey company, Credamo, as [63] pointed out that there were no statistical differences between the results of face-to-face questionnaires and email questionnaires. The target respondents were selected based on their habits of eating potato chips or red date walnuts because these types of buyers would pay more attention to the attributes of the foods [64]. In October 2020, a total of 570 respondents participated in the online survey, of which 300 in the red date walnut group, and 270 in the potato chips group. Respondents could only answer one of the two questionnaires. Each questionnaire consisted of four parts: (1) anonymous information (gender, age, weight, and height) about the respondents, (2) the FTP scale, (3) cognition about the health level of the given food product, and (4) the selection of the choice sets in the CE.

We set a “attention check” in the questionnaire to identify the careless respondents [65]. The question was “Please choose ‘red’ from the following options.” If other colors were selected, the questionnaire would be identified as invalid.

### 2.4. Models

The CE was based on the Lancaster consumer theory [66], which states that the consumer utility is derived from the attributes of the product rather than the product itself. It can be expressed mathematically as:

*U_nit_ = V_nit_ + ε_nit_* (1)

An individual *n*’s utility from alternative *i* is expressed as *U_nit_*. The consumer utility consists of the observable representatives *V_nit_* and the unobservable random error *ε_nit_* [67].

In discrete choice modeling, different hypotheses of the random error distribution and heterogeneity will lead to different models. In this study, we assumed that all respondents share a homogeneous preference for the attributes of the product [49], and thus the multinomial logit (MNL) model was adopted, which is the basic form of logit modeling.

When estimating the main effects of the attributes, consumer utility can be expressed with Equation (2). In this study, the attribute “Label” is a nominal variable, as it represents the different forms of labels. Dummy variables were generated with the baseline of not being labeled. The price is the metric variable, and was designed with the four levels shown in Table 1.

*U_nit_ = β_1_Price_nit_ + β_2n_Kcal_nit_ + β_3n_Walk_nit_ + β_4n_Run_nit_+ + ε_nit_* (2)

The *nit* of each variable indicates the attributes for which individual *n* chooses an alternative *i* in the choice set *n. β*_1_ to *β*_4n_ are the parameter vectors of the attributes to be estimated.

As for the interaction effects between the attributes and FTP, we put the FTP variable into Equation (3). FTP is a metric variable, and *FTP_n_* is the mean score of individual *n*’s answers to the five questions. *β*_1_ to β_7n_ are the parameter vectors of the attributes to be estimated:

*U_nit_ = β_1_Price_nit_ + β_2n_Kcal_nit_ + β_3n_Walk_nit_ + β_4n_Run_nit_ + β_5n_(Kcal_nit_*
× *FTP_n_*) *+ β_6n_(Walk_nit_ × FTP_n_) + β_7n_(Run_nit_ × FTP_n_) + ε_nit_* (3)

Individual *n*’s WTP for attributes *x* is estimated as:

(4) $${WTP}_{n}\;=\;\frac{\beta nx}{\beta np}$$

where *β_nx_* is the coefficient of the non-price attribute *x* and *β_np_* is the coefficient of the price attribute *np*.

## 3. Results

### 3.1. Sociodemographics of Consumers

After dropping the questionnaires containing careless answers to the “attention check”, there were 285 questionnaires left in the red date walnut group and 243 in the potato chip group. All of the data are calculated with Stata 15.0. Similar distributions could be found in the two groups. Female respondents slightly exceeded men in both groups, making up 56.49% and 53.50%, respectively. Among all of the respondents, ages between 25 and 34 had the highest percentages in both groups (69.93% and 67.90%), followed by people under 24 years old and 35–44 years old. However, respondents over 45 years old were rare in this study. We also asked respondents to fill in their weight and height to calculate the body mass index (BMI) of the respondents. The mean BMI scores were 21.32 in the red date walnut group and 20.90 in the potato chip group (Table 2).

All item scores for the FTP are shown in Table 3. All of the respondents showed mean scores of more than 4 for five of the questions. The results indicated that the respondents in this study had higher levels of FTP. In the test of the consumers’ cognition of the health levels of the two products, a Pearson correlation analysis is used to observe the degree of correlation between two items. The results of correlation analysis showed that the correlation between two items in two products was significant (*p* < 0.001), which indicated that the two items concerning the health level were significantly correlated and thus reliable to form a health level index. The mean scores for the health levels demonstrated an obvious difference between the two groups. The mean value was 4.04 in the red date walnut group, which means that the consumers recognized red date walnuts as a healthy food. Meanwhile, the score for potato chips was 2.78 (below the average of 3.5 on the five-point Likert scale), which indicated that people perceived that potato chips contain few nutrients. These results proved that the foods we chose in this study were able to represent an unhealthy and a healthy alternative.

### 3.2. Main Effects

The main effects of the standard calorie and PACE labels are illustrated in Table 4. All of the attributes were statistically significant, which implies that the labels in this study had effects on the consumers’ food preferences. In the red date walnut group, all coefficients of the labels and price were statistically significant at the 1% level. Compared with not using a label, providing information about the calories contained in the red date walnuts can enhance the consumer utility. Of the three forms of labels, people showed the most positive attitudes toward the PACE labels, including labels showing the minutes of walking and minutes of running, followed by the kcal label. The coefficient of the price variable was significantly negative at the 1% level, which means that a higher price would attenuate the consumers’ preference for the product. As for the potato chip group, all results showed a completely opposite phenomenon. Compared with not using a label, displaying any information about the calories of the potato chips has a negative influence on the preferences for the product; people had stronger negative preferences for the minutes of walking label, as well as the kcal label. Increasing the price was also able to undermine the consumer utility of the potato chips. In general, for the healthy alternative, people preferred to be provided information about calories, especially with PACE labels, indicating that PACE labels may improve consumers’ preferences for a product and promote the purchase intention. Conversely, the calorie and PACE labels reduced the purchase intention for the snack product.

### 3.3. Main Effect Including the Interaction with the FTP

Taking the FTP into consideration, the results of the main effect including the interaction are shown in the Table 4. The coefficients of most attributes remained robust after interaction with FTP. In the red date walnut group, the results were statistically significant at the 5% level, and the coefficients of the conjoint variables were positive which means that people with higher FTP scores were more inclined to accept calorie and PACE labels and showed positive preferences for those labels on healthy products. The likelihood of buying red date walnuts with the minutes of walking label was the highest, following by the product with the kcal label and the minutes of running label. In the potato chip group, the coefficients of the PACE labels were significantly negative at the 5% level. This indicated that people with higher FTP scores were less likely to buy unhealthy products with PACE labels, especially with the minutes of running label (−0.57). In addition, the preference for the kcal label was not significant when considering the FTP, which implies that there was no difference between not having a label and the kcal label for people with higher FTP scores.

### 3.4. Interaction between the Main Effect and Sociodemographics

Results of interaction effects between labels and socio-demographics were shown in Table 5. In the red date walnut group, gender × kcal label and gender × running label showed significantly negative effects, indicating that women were reluctant to choose red date walnuts with the kcal and running labels. Compared with the exercise-display form, only the kcal label was able to undermine the evaluations of the food product by female consumers. This might be because women pay more attention to the calories in foods [68]; a healthy food with many calories may attenuate consumers’ preferences for the health attribute. Younger consumers showed less preference for the running label, which might be because younger consumers show negative attitudes toward long periods of exercise. The coefficient of BMI × kcal label was significantly negative at the 5% level, which indicates that consumers with higher BMI did not intend to choose walnuts with the kcal label. The potential reason is that a healthy food with many calories is opposite to the healthy goals, and directly displaying the calorie information may generate negative preferences for healthy food products. As for chips, BMI × walk label was significantly positive, which means that a person with a higher BMI was more likely to choose chips with the minutes of walking label. This might be because people are inclined to burn off calories in a moderate way (walking).

### 3.5. WTP

Table 6 demonstrated the WTP for different labels. In the red date walnut group, the consumers showed positive preferences for the calorie and PACE labels, and the WTP for PACE labels with walking and running was 2.78 and 2.75 RMB higher, respectively and the WTP for the kcal label was about 1.90 RMB higher. In the chip group, the consumers discounted the chips when calorie and PACE labels were provided, and indicated a WTP of 0.288 RMB for the walking label, 0.222 RMB for the kcal label, and 0.207 RMB for the running label. This indicates that the PACE and kcal labels can decrease the purchase intention for chips.

## 4. Discussion

In this study, the effects of four labels (none, standard kcal label, minutes of walking to burn off the calories, and minutes of running to burn off the calories) on consumers’ preferences for two products (red date walnuts and potato chips) were explored with a CE approach, and we also estimated the interaction effects between FTP and socio-demographic variables. Based on the results of this study, three important research conclusions and their theoretical contributions are summarized.

Firstly, the effects of the PACE labels, standard calorie label, and lack of label on consumers’ preferences were investigated. Results showed that PACE labels have statistically significant effects on consumers’ preferences for both products in this study. It has also been demonstrated that the public prefer PACE labels over other types of food labels [69]. The significant effects of PACE labels on consumers’ preferences confirmed the effectiveness of PACE labels and calorie label on food preferences in previous studies [27], also indicating that PACE labels are easier to understand so that consumers can determine whether these calories are “worth” consuming [23].

Results of this study illustrated that the coefficients of minutes to walk label (0.54) and minutes to run label (0.54) were higher than the standard calorie label (0.37) in red date walnut group, which means PACE labels can most effectively improve consumers’ preferences for healthy food. However, the effects of those three labels were changed in potato chips group. Minutes to walk label (−0.25) has a more negative effect on consumers’ preferences for unhealthy food than the calorie label (−0.20), while minutes to run label (−0.18) was inferior to the calorie label. In previous studies, investigators showed the effects of PACE labels from multiple perspectives, such as by researching the utility of PACE labels from the perspective of consumers’ sensory and emotional cognitions [45], studying the applicable conditions of PACE labels from the perspectives of high-energy and low-energy food types [26], studying the effects of PACE labels on consumers’ food consumption and post-consumption movement [44], or studying the moderating effect of consumers’ health concerns on the preference for PACE labels [62]. However, no known research has specifically distinguished between the exercises shown on PACE labels or explored the differences in the effects when showing different exercises. It is believed that the calories may be displayed in the form of different exercises, such as slow walking or fast running, on the PACE labels. The research results suggested that exploration of different exercises shown on PACE labels is necessary. In this study, the effects of the minutes of walking label on both healthy food and unhealthy food were significantly better than those of the minutes of running label, indicating that a walking label showing a longer time can initiate stronger preferences for healthy food as well as the effect of control over indulgent consumption. In the future, how the movement and time dimensions of PACE labels can be used to better optimize PACE labels and the factors that cause consumers to prefer longer times (walking) instead of more intense movement (running) can be more deeply explored.

Secondly, the results showed that compared with the no-label condition, the positive or negative effects of the PACE and calorie labels on consumers’ preferences depending on the food type. In this study, for the healthy alternative (red date walnut), compared with the lack of a label, the PACE and calorie labels significantly improved preferences of consumers, and consumers in this group preferred PACE label with minutes to walk most. For the unhealthy food (potato chips), the main effects of the labels were the absolute opposite, which means that people were reluctant to see any information about the calories contained in the potato chips, especially with the label showing the minutes of walking needed to burn off the calories. In this group, the PACE and calorie labels significantly reduced preferences of consumers. The contrary results were in line with previous literature that effects of FoP labels on consumers preferences were inconsistent between healthy and unhealthy foods [31,32,33,69]. The potential reason for the contrary results is due to the consumers’ anticipatory guilt about high-calorie foods [45,70]. In other words, the guilt that comes from indulgences like unhealthy is hard to justify, so that PACE labels exert a strong restrained effect on such foods. As for healthy foods, eating healthy foods may become a justification for consumers to ignore the negative aspects of the foods (i.e., high-calorie content) [71].

Thirdly, the future time perspective (FTP) [40] was introduced to further analyze the interaction effects of the labels in this study. It was found that the FTP can strengthen the positive influence of PACE labels on consumers’ preferences for healthy food as well as the inhibition of PACE label of consumers’ preferences for unhealthy food, which was consistent with our expectations. On one hand, as a characteristic of consumers’ ability to behave in consideration of the future [72], FTP can inhibit consumers’ preferences for unhealthy food through PACE labels by improving the ability to delay gratification [39]. Furthermore, consumers with high FTP are more likely to use the information contained in the PACE labels to activate their anticipatory guilt about unhealthy high-calorie foods, thereby reducing the likelihood of potential guilt by avoiding the purchase of this type of food [69]. On the other hand, FTP can strengthen the effects of PACE and calorie labels on their preferences for healthy food. This also confirmed that consumers with high FTP have higher subject awareness of health [40], and this may also be the result of consumers with high FTP considering the benefits of healthy food in the future [73]. Meanwhile, healthy foods with PACE labels serve as a visual information that allows consumers with high FTP to have more self-control over their caloric intake. Although such healthy foods are high in calories, the health benefits and caloric control justify [68] reducing the threat of high calories. In this study, FTP not only improved the applicability of the interaction effect in terms of consumers’ characteristics, but also provided an insight into the potential effects of PACE and calorie labels on consumers’ preferences for food [74]. It provided a new psychological perspective of consumers that can be interesting for future research on PACE labels, and it echoes the aforementioned possibility that due to time pressure and sensitivity, PACE labels showing different exercises have different interaction effects.

Fourthly, the socio-demographic of respondents (age, gender, BMI) were contained in relative literature about PACE labels [16,36]. We found that women (vs. men) were less inclined to choose health food with calorie labels and minutes to running label, and the elderly (vs. younger) people in the healthy food group preferred minutes to running label. People with a higher BMI were reluctant to purchase walnuts with minutes to walk label. For other sociodemographic variables, no significant interaction with labels was found in other studies [14,59], so they were not included in the study for analysis.

### 4.1. Theoretical Contribution and Practical Implication

This study has two important theoretical contributions: The first theoretical contribution of this study is to demonstrate the difference in the effects of PACE labels on different food types of varying healthiness, but also tried to explain the current literature conflict in the study of effects of PACE labels. Meanwhile, because PACE labels can improve consumers’ preferences for healthy food, but inhibit consumers’ preferences for unhealthy food [23]. In real market, consumers may be encouraged by PACE labels to purchase healthy food instead of unhealthy food [16,75]. And this shift in consumption patterns due to the PACE label has theoretical significance for other scholars who study FoP labels.

The second contribution is to determine the interaction effects between FTP and PACE labels. No previous study has analyzed the relation between FTP and food choices in the context of PACE labels. FTP in this study not only improved the applicability of the interaction effect in terms of socio-demographics, but also had an insight into the potential effects of PACE and calorie labels on consumers’ preferences for different type of foods. In future research, efforts should not only put on the interactive analysis of labels and socio-demographics, but also introduce a psychological perspective (such as time perspective, self-efficacy, etc.) to analyze consumers’ preferences for foods.

The results of this study can also give some practical implications to the government, health organizations and food enterprises. As a visual reminder of calorie information, PACE labels can be used as a nudge tool to effectively improve consumer behavior [9,10]. And we’ve also shown that it’s a much stronger boost than numerical calorie labels [23], thus, it is necessary to improve consumers’ preferences for healthy food through effective marketing means [75]. To be specific, the PACE labels can be actively used to promote the purchase of healthy foods. Meanwhile, in order to improve the promotion effect of PACE label on healthy food, we should focus on the specific types of PACE label in more detail. Therefore, choosing minutes to walk label as the form of PACE labels can better promote the purchase of healthy food, which is also in line with the thought of nudge strategy [76]. Therefore, for enterprises producing high-calorie healthy foods, they should attach the PACE labels to the food to promote consumer preferences for the product. At the same time, snack makers may have little incentive to push nudges such as PACE labels, which can reduce consumer preferences for unhealthy foods that are high in calories. For promoting healthy food consumption, governments and health organizations should develop industry regulations that require such enterprises to have the responsibility and obligation to attached the PACE labels.

### 4.2. Limitations and Future Research Directions

The limitations and future research directions of this study are as follow. Firstly, in this study, a CE was used to explore the main effects of PACE labels, with no labeling and calorie labeling, and the moderating effect of healthy and unhealthy food types on this main effect was also explored. But what psychological factors of consumers ultimately lead to the main and moderating effect? This is not explored in this article. Although in the discussion, we tried to explain it through consumers’ anticipatory guilt and justification reasons, in the future research, we still need to analyze it through more rigorous behavioral experiments and explore the stabilizing role of the potential psychological mechanism in the main and moderating effect.

Secondly, we choose age, gender and BIM as the basic socio-demographics to analyze the main effects of PACE label and calorie label, and obtained a certain result of interaction effect in this study. At the same time, we also introduced FTP as a consumer characteristic to analyze the interaction effect. However, there are also many consumer characteristics that deserve to be studied, such as time pressure and sensitivity [77], which may affect consumers’ sensitivity to calorie information. For example, an individual with high time pressure may avoid high-calorie foods [78], both healthy and unhealthy, because he or she has no time to burn calories. What’s more, an individual with high time pressure may need high calories as fuel to improve his or her job performance [79]. In addition, perceived pleasantness also may have influence on healthy behaviors [80]. Therefore, as an important feature of contemporary people, time pressure and sensitivity are worth studying and coordinating the contradictions between different research conclusions.

## 5. Conclusions

This study demonstrates that PACE labels have different influences on consumers’ preferences for difference food types. In terms of red date walnuts, which were the representative healthy food in this study, people showed positive preferences for products with PACE labels. In other words, healthy foods with PACE labels are more likely to be purchased by consumers. On the other hand, consumers showed a negative preference for potato chips with kcal and PACE labels, which means that people are reluctant to buy unhealthy foods with those labels, especially with PACE labels that show information on the minutes of walking needed to burn off the calories. FTP was shown to enhance the preferences for healthy food with PACE labels, while people with higher FTP scores showed negative preferences for unhealthy food with PACE labels. This study indicates that PACE labeling may be an effective strategy for promoting purchase of healthy foods, as well as a tool for decreasing purchase intentions for unhealthy food.

## Figures and Tables

**Table 1:** Product attributes and levels in the choice experiment.

Attributes	Number of Levels	Levels
Label	4	None, kcal label, minutes of walking label, and minutes of running label
Price	4	22.60, 24.90, 27.40, and 30.00 RMB (for red date walnuts)
		5.60, 6.50, 7.20, and 7.80 RMB (for potato chips)

Note: RMB = Chinese yuan; 1 US dollar = 6.43 RMB.

**Table 2:** Sociodemographics of respondents.

Red Date Walnut				Potato Chips			
Variable	Definitions	Frequency	Percentage	Variable	Definitions	Frequency	Percentage
Gender	Male	124	43.51%	Gender	Male	113	46.50%
	Female	161	56.49%		Female	130	53.50%
Age	≤24 years old	45	15.73%	Age	≤24 years old	56	23.05%
	25–34 years old	200	69.93%		25–34 years old	165	67.90%
	35–44 years old	34	11.89%		35–44 years old	18	7.41%
	45–54 years old	7	2.45%		45–54 years old	3	1.24%
	55–64 years old	0	0		55–64 years old	0	0
	≥65 years old	0	0		≥65 years old	1	0.41%
BMI: mean	21.32			BMI: mean	20.90		

**Table 3:** Scores for the future time perspective (FTP) and health levels.

Red Date Walnut			Potato Chips		
		Mean (SD)			Mean (SD)
FTP	Mean	4.14(0.50)	FTP	Total	4.20(0.47)
	α	0.71		α	0.71
Health level	Mean	4.04(0.59)	Health level	Mean	2.78(0.96)
	r	0.46		r	0.73
	*p* < 0.001			*p* < 0.001	

**Table 4:** Estimation of the direct effects and interaction effects of the labels using the MNL models.

	Main Effect		Main Effect with Interaction	
Variables	Red date walnut	Potato chips	Red date walnut	Potato chips
Kcal label	0.37 ***	−0.20 **	−1.34 *	0.48
(SD)	(4.08)	(−2.00)	(−1.84)	(0.54)
Minutes to walk label	0.54 ***	−0.25 **	−1.64 **	1.90 *
(SD)	(5.42)	(−2.29)	(−1.98)	(1.87)
Minutes to run label	0.53 ***	−0.18 *	−1.08	2.19 **
(SD)	(6.01)	(−1.83)	(−1.46)	(2.45)
price	−0.20 ***	−0.88 ***	−0.20 ***	−0.89 ***
(SD)	(−14.01)	(−14.53)	(−14.03)	(−14.52)
FTP × Kcal label			0.42 **	−0.16
(SD)			(2.37)	(−0.76)
FTP × walk label			0.53 ***	−0.51 **
(SD)			(2.65)	(−2.14)
FTP × run label			0.39 **	−0.57 ***
(SD)			(2.21)	(–2.67)
Log likelihood	−1158.85	−973.05	−1154.54	−968.92
Prob > chi2	0.00	0.00	0.00	0.00
Observations	3820	3240	3820	3240

Note: *, **, and *** indicate significance at the 10%, 5%, and 1% levels, respectively.

**Table 5:** Estimation of the interaction effects of the calorie labels with the sociodemographic variables using the MNL models.

	Main Effect with Interaction	
Variables	Red date walnut	Potato chips
kcal label	2.58 ***	−1.91 *
(SD)	(2.81)	(−1.66)
walking label	3.49 ***	−1.69
(SD)	(3.22)	(−1.31)
running label	2.55 ***	−0.13
(SD)	(2.62)	(−0.11)
price	−0.20 ***	−0.88 ***
(SD) (SD)	(−14.00)	(−14.51)
gender × kcal label	−0.43 **	0.04
(SD)	(−2.24)	(0.20)
gender × walking label	−0.24	−0.23
(SD)	(−1.07)	(−0.93)
gender × running label	−0.34 *	−0.10
(SD)	(−1.69)	(−0.46)
age × kcal label	−0.00	0.01
(SD)	(−0.03)	(0.29)
age × walking label	−0.04 *	−0.01
(SD)	(−1.85)	(−0.56)
age × running label	−0.01	−0.02
(SD)	(−0.70)	(−1.01)
BMI × kcal label	−0.07 **	0.07
(SD)	(−2.02)	(1.52)
BMI × walking label	−0.07	0.10 **
(SD)	(−1.64)	(1.98)
BMI × running label	−0.05	0.03
(SD)	(−1.39)	(0.67)
Log likelihood	−1150.68	−966.98
Prob > chi2	0.00	0.00
Observations	3820	3240

Note: *, **, and *** indicate significance at the 10%, 5%, and 1% levels, respectively.

**Table 6:** Willingness to pay (WTP) for different calorie labels.

Attributes	Red Date Walnuts		Potato Chips	
	Mean (RMB)	CI	Mean (RMB)	CI
		[5%, 95%]		[5%, 95%]
Kcal label	1.90	[1.027, 2.772]	−0.22	[−0.45, 0.00]
Minutes of walking label	2.78	[1.699, 3.852]	−0.29	[−0.53, −0.05]
Minutes of running label	2.75	[1.751, 3.750]	−0.21	[−0.43, 0.00]

## Data Availability

The authors confirm that the datasets analyzed during the study are available from the first au-thor or the corresponding author upon reasonable request.
